# An Energy Efficient Synchronization Protocol for Target Tracking in Wireless Sensor Array Networks

**DOI:** 10.3390/s19061367

**Published:** 2019-03-19

**Authors:** Jie Shen, Ming Yin, Ji-An Luo, Zhi-Bo Wang, Zhi Wang, Zhen-Hui Li

**Affiliations:** 1 Department of Control Science and Engineering, Zhejiang University, Hangzhou 310027, China; zjushenjie@126.com (J.S.); yinming1026@zju.edu.cn (M.Y.); wangzhizju@gmail.com (Z.W.); 2 Key Lab for IOT and Information Fusion Technology of Zhejiang, Hangzhou Dianzi University, Hangzhou 310018, China; luojian@hdu.edu.cn; 3 School of Cyber Science and Engineering, Wuhan University, Wuhan 430072, China; zbwang@whu.edu.cn; 4 Advanced Technology Institute, Zhejiang University, Hangzhou 310027, China

**Keywords:** synchronization, target tracking, sensor arrays, wireless sensor networks

## Abstract

Time synchronization is an important middleware function that supports the Quality of Service (QoS) of systems in wireless sensor array networks. Instead of providing high synchronization accuracy for all application scenarios, we argue that synchronization protocols should be application specific. In this paper, we exploit the synchronization requirements of target-tracking systems in wireless sensor array networks and propose an energy-efficient Sensor Array Synchronization Protocol (SASP), which provides the required synchronization accuracy to guarantee the QoS. Specifically, when no target appears, to guarantee system lifetime, coarse synchronization is achieved with little overhead by piggybacking time information onto periodical network maintenance packets. Once targets appear, SASP achieves high inter-array and relatively higher intra-array synchronization accuracy rather than the traditional network-wide high accuracy on average. In this way, it guarantees reliable communication and accurate data fusion, while reducing energy consumption. Theoretical analysis and extensive evaluations show the effectiveness of the proposed protocol.

## 1. Introduction

Wireless Sensor Networks (WSNs) [1] have been widely used in many applications, such as environmental monitoring [2,3], localization [4,5], data publishing with privacy [6,7], target tracking [8,9], and healthcare [10,11]. In many cases, WSNs are large-scale and decentralized with thousands of nodes [12]. Time synchronization, which builds a common understanding of time among all nodes, is one of the most important techniques in WSNs [13,14,15] and other wireless scenarios [16,17,18]. However, due to the low quality of crystal oscillations, the limited computation capabilities, the uncertainty in wireless communication, and the limited power supply, time synchronization in WSNs is still a challenging problem.

In recent years, many synchronization protocols for WSNs have been proposed. Most of them focus on achieving high synchronization accuracy, e.g., at tens of µs or even higher clock accuracy level. However, higher accuracy is usually at the cost of larger energy consumption, which significantly reduces the lifetime of sensor nodes and also sensor networks. In practice, different systems have different time synchronization accuracy requirements, and it is not necessary for the sensor network to provide high synchronization accuracy for all the systems. For example, a few systems [19,20] could operate well with millisecond (ms)-level clock accuracy. Moreover, one system may have different synchronization accuracy requirements in different situations. For example, let us consider a target-tracking system that is deployed in the wild to detect non-cooperative targets. When no target appears, the system works in sleep mode, and *coarse synchronization* is required to wake nodes up simultaneously for periodical network maintenance. However, once a target appears, nodes surrounding the target should sense it and report their sensing results to the sink node for data fusion [21]. In order to realize reliable communication and accurate data fusion, *precise synchronization* is required. Therefore, instead of designing synchronization protocols with high accuracy, we argue that *synchronization protocol design should be application specific*.

In this paper, we focus on the synchronization protocol designed for a target-tracking system in wireless sensor array networks. Note that a wireless sensor array network is a special type of WSN that organizes nodes into arrays to finish tasks. We propose an energy-efficient synchronization protocol called the *Sensor Array Synchronization Protocol* (*SASP*) to satisfy the different synchronization requirements and also reduce energy consumption. Specifically, we first analyze the effects of synchronization accuracy on the Quality of Service (QoS), like system lifetime, communication delay, and localization accuracy. For each QoS, given the tolerable value, the required (or tolerable) synchronization accuracy is obtained correspondingly. The requirements have two notable features: (a) The required accuracy in the detection mode is higher than that in the sleep mode. (b) The intra-array accuracy is required to be higher than the inter-array accuracy in the detection mode. Thus, SASP achieves different levels of accuracy according to the system mode and the network scope, i.e., coarse synchronization in the sleep mode, and high inter-array and relatively higher intra-array synchronization in the detection mode. In this way, we satisfy the synchronization requirements, while reducing the energy consumption. The contributions of this paper are summarized as follows:

- We propose that time synchronization can (or should) be optimized for the specific application that executes on the wireless sensor network. This idea can be extended to other protocols and systems.
- We thoroughly analyze the synchronization accuracy requirements for a target-tracking system in wireless sensor array networks and study the effects of synchronization accuracy on the QoS of the system.
- We propose an energy-efficient synchronization protocol that satisfies the different synchronization accuracy requirements with the minimum energy consumption.
- We conduct simulations to evaluate the effectiveness of our synchronization protocol.

The rest of the paper is organized as follows. Section 2 discusses the related work for time synchronization. Section 3 briefly introduces our system and presents the problem formulation. In Section 4, we analyze the synchronization accuracy requirements in detail. The design of SASP is presented in Section 5. We evaluate SASP in Section 6. Finally, Section 7 concludes the whole paper and plans the future work.

## 2. Related Work

In recent years, researchers have proposed many time synchronization protocols for WSNs. *TPSN* (Timing-Sync Protocol for Sensor Networks) [22] applies the two-way communication scheme, i.e., the sender transmits time information to the receiver, then the receiver transmits information back, to achieve precise pair-wise clock synchronization. *RBS* (Reference Broadcast Synchronization) [23] uses a receiver-receiver scheme, which broadcasts a synchronization message and compares the message received time of each receiver to eliminate random delays on the sender side. *FTSP* (Flooding Time Synchronization Protocol) [24] achieves high synchronization accuracy by time-stamping a broadcast message multiple times, on both the sender and receiver. These protocols mainly focus on how to transmit time information to achieve better accuracy, e.g., *RBS*, *TPSN*, and *FTSP* achieve accuracies of 29.1 µs, 16.9 µs, and 1.4 µs, respectively. However, high accuracy is usually at the cost of large energy consumption. Because of the limited power supply in WSNs, time synchronization should be studied together with energy saving. Zhong et al. [25] developed a clock uncertainty-driven mechanism to minimize communication overhead and provide the desired accuracy. Xie et al. [26] proposed a novel algorithm using a fast finite-time average consensus idea. Brunelli et al. [27] presented a low-overhead clock synchronization method based on a temperature compensation algorithm. However, solutions like *SASP*, which is based on the features and actual requirements of a real system, have rarely been proposed.

At the same time, several well-known systems have been developed on which time synchronization techniques are used. *VigilNet* [8] uses a method that broadcasts the synchronization beacon frame from base station periodically to guarantee synchronization accuracy. *PinPtr* [19] employs *FTSP* to synchronize the nodes’ clocks. Although these systems work well, we argue that the employed synchronization protocols can (or should) be further optimized by the accuracy requirements of the systems. For example, the experiments of *PinPtr* show that the synchronization accuracy achieved by *FTSP* (1.4 µs) is much better than the accuracy required by the system (0.5 ms). Since achieving 1.4 µs consumes more energy than achieving 0.5 ms, *PinPtr* can optimize its synchronization protocol to save energy.

In this paper, we exploit the synchronization requirements of the target-tracking system. Based on the requirements, we propose *SASP*. *SASP* does not aim to achieve high accuracy all the time, but to achieve variable accuracies according to the requirements to save energy.

## 3. System Introduction and Problem Formulation

An acoustic sensor array is a group of sensors, usually deployed in a certain geometric pattern, that collect and process acoustic signals for relevant parameter estimation. Sensors belonging to the same array often receive identical source signals, except with a phase shift due to the time difference of the plane wave-front at different sensors. Thus, the Direction of Arrival (DoA) of sources can be estimated using the time differences within an array. Further, triangulation source localization can be realized by at least two bearing measurements (DoA).

**Figure 1:**
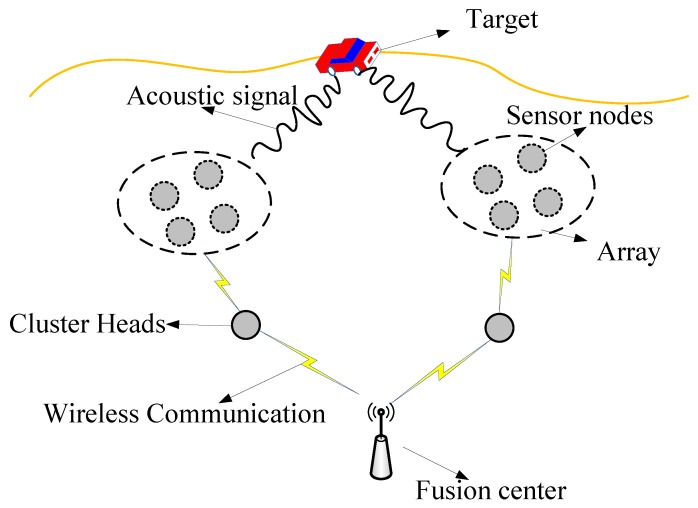
Illustration of the target-tracking system.

In the traditional sensor array systems [9,28,29,30], sensor nodes are connected by wires to form arrays. There is a local processor in each array to perform DoA estimation. The estimated results (only several bytes) are wirelessly transmitted to the fusion center. However, these systems have the following drawbacks: First, wired connections are costly in deployment and maintenance. Second, the local processors are usually energy consuming and expensive. To address the drawbacks, we execute our target-tracking system on wireless sensor array networks. As shown in Figure 1, the wireless sensor nodes are organized into arrays based on their location information. We have the following definitions:

- **Intra-array nodes**: nodes in the same array.
- **Inter-array nodes**: nodes of different arrays.

Each sensor node is equipped with a microphone to detect the acoustic signal. We use compressive sensing [31], a random sampling and deep quantization technique, to reduce the amount of sensing data significantly. All the raw sensing data are sent to the fusion center using wireless communication.

In WSNs, the wireless communication module is one of the most energy-consuming modules, which consumes more than 90 percent of the energy [32]. Hence, to prolong the lifetime of a system, it is necessary to reduce the energy consumption of wireless communication.

In order to guarantee high communication quality with predictable performance, the TDMA (Time Division Multiple Access) protocol is adopted in our system. The network topology of our system is a three-level tree topology. As shown in Figure 1, we have one sink center, several cluster heads, and many sensor nodes. Each sensor node can communicate with its cluster head, and all the cluster heads can communicate with the sink center. Each cluster head manages the TDMA slots of its child sensor nodes. The sink center manages the TDMA slots of all the cluster heads.

When there is no target in the area of interest, the communication module is turned off, and the system works in the *sleep mode* to prolong the lifetime. However, the network system needs to wake up periodically to transmit packets for network maintenance. If the clocks are not well synchronized, nodes cannot wake up at the same time. In this situation, each node’s awake time is prolonged to guarantee reliable transmission of network maintenance packets, which consumes more energy and reduce the system’s lifetime. Thus, time synchronization is essential for prolonging the system’s lifetime.

When a target appears, the system works in the *detection mode*. Nodes that detect the target should send their sensing data to the fusion center. However, TDMA requires the nodes to have a common notion of time, otherwise, data collision will occur. Thus, reliable data communication requires time synchronization.

After sensing data are transmitted to the fusion center, data fusion is performed to estimate the location of the target, which contains the following two steps (details are described in Section 4):

- **Intra-array fusion:** processing the detection data and getting the acoustic arrival time to each sensor node of an array, we can figure out the direction of the target to the array by comparing the arrival time.
- **Inter-array fusion:** using direction information from several arrays, we can calculate the target’s location.

Since the target is moving all the time, if the clock of each sensor node is not synchronized, the system cannot determine the location of the target because it does not know whether the detections from different sensor nodes are performed at the same time for the same location of the target. Therefore, in order to guarantee accurate intra-array and inter-array fusion, we have:

- **Intra-array synchronization**: synchronize the nodes in the same array.
- **Inter-array synchronization**: synchronize the nodes of different arrays.

In short, to guarantee the QoS of the system, e.g., system lifetime, reliable communication and accurate data fusion, time synchronization is required. However, time synchronization brings a negative effect, i.e., extra energy consumption. Furthermore, achieving higher synchronization accuracy consumes more energy. Thus, this demands a tradeoff between synchronization accuracy and energy efficiency. In this paper, we aim to design a synchronization protocol to satisfy the accuracy requirements of the target-tracking system, while minimizing the energy consumption. The problem is formulated as:

(1) $$\begin{array}{cc} \mathrm{minimization} & E_{syn},\\ \mathrm{subject}\;\mathrm{to} & 0<\delta \leq \delta_{i},i=1,2,\ldots ,m. \end{array}$$

where $E_{syn}$ is the energy consumption of time synchronization; δ is the achieved accuracy, where smaller δ means smaller error between clocks, i.e., higher accuracy; $\delta_{i}$ denotes the required (tolerable) accuracy; and *m* denotes the number of the accuracy constraints.

$E_{syn}$ mainly contains two parts: communication consumption and computation consumption, the later one being negligible compared with the former one [12]. Thus, we regard $E_{syn}$ as the energy of transmitting synchronization packets. If we synchronize the network more frequently, more packets are transmitted, which consumes more energy, but we can achieve higher synchronization accuracy. Thus, $E_{syn}$ is directly related to the achieved synchronization accuracy δ. We use a function f(*) to denote the relationship between $E_{syn}$ and δ, having $E_{syn}=f\left(\delta \right)$, where f(*) is a monotonically-decreasing function [33]. However, the mathematical expression of f(*) is difficult to obtain, since it depends not only on the synchronization scheme, but also on the hardware, the environments, etc.

The purpose of transmitting synchronization packets is to send synchronization information. However, if we can send synchronization information with the data packets, the synchronization packets are not required. This strategy is called *piggybacking*. Let $I_{pig}$ denote piggybacking; if synchronization information is sent by piggybacking, then $I_{pig}=0$, otherwise, $I_{pig}=1$. Overall, $E_{syn}$ is modeled as follows:

(2) $$E_{syn}=\left\{\begin{array}{c} f_{1}\left(\delta \right),I_{pig}=0\\ f_{2}\left(\delta \right),I_{pig}=1 \end{array}\right.,$$

where $f_{1}$ denotes the relationship between $E_{syn}$ and δ when synchronization information is sent by piggybacking, and $f_{2}$ denotes the relationship between $E_{syn}$ and δ when synchronization information is not sent by piggybacking.

## 4. Accuracy Requirements Analysis for the Target-Tracking System

### 4.1. Demand of Sleep Mode

In order to prolong the network’s lifetime, our system works in the sleep mode when no target appears. However, sensor nodes need to wake up periodically for network maintenance. The duty ratio *D* is defined as the percentage of awake time every period. Suppose that energy supplied for the sleep mode is $E_{s}$. Nodes work in the receiving (idle) or sending state when the system is awake. The energy consumption of the receiving and sending state is almost the same in our system, which is denoted by $e_{1}$. Nodes work in the sleep state when the system is asleep, and the energy consumption is $e_{2}$. We can calculate node lifetime *L* as:

(3) $$L=\frac{E_{s}}{e_{1}D+e_{2}\left(1-D\right)}.$$

Under ideal situation without clock error, nodes will wake up simultaneously to send and receive network maintenance packets. Therefore, the node’s duty ratio is determined by the operation period *P* and the time for network maintenance packet transmission $T_{tr\_m}$, and we have:

(4) $$D_{ideal}=\frac{T_{tr\_m}}{P}=\frac{l_{m}}{rP},$$

where $l_{m}$ denotes the length of the network maintenance packet and *r* denotes the sending rate.

**Figure 2:**
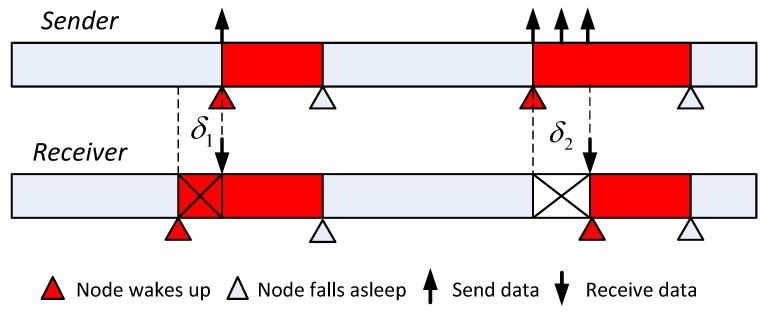
Send and receive network maintenance packets in the sleep mode.

However, clock error between the sender and the receiver exists in real situations. As shown in Figure 2, because of the clock error, it would wake up $\delta_{1}$ earlier and keep listening. Alternatively, the sender node could prolong the sending time window (i.e., re-transmission). Both strategies will consume extra energy. Suppose that the clock error is $\delta_{s}$ ($\delta_{s}>0$) and receivers wake up $\delta_{s}$ earlier to guarantee a reliable transmission. Therefore, the duty ratio changes to:

(5) $$D=\frac{T_{tr\_m}+\delta_{s}}{P}=\frac{l_{m}+r\delta_{s}}{rP}.$$

Combining (3) and (5), we get the node’s lifetime with clock error as:

(6) $$L=\frac{rE_{s}P}{e_{1}l_{m}+re_{1}\delta_{s}+re_{2}P-e_{2}l_{m}-re_{2}\delta_{s}}.$$

In many cases, the system lifetime is designed to be greater than the individual sensor’s lifetime by deploying redundant nodes. However, in this paper, the system lifetime is assumed to be equal to the node lifetime for simplicity.

In order to accomplish long-term missions, the system lifetime should be longer than the expected lifetime $L_{d}$, i.e., $L\geq L_{d}$, so the synchronization accuracy in the sleep mode should satisfy:

(7) $$0<\delta_{s}\leq \frac{rE_{s}P-e_{1}l_{m}L_{d}-re_{2}PL_{d}+e_{2}l_{m}L_{d}}{re_{1}L_{d}-re_{2}L_{d}}$$

### 4.2. Demand of the Communication Protocol

When targets appear, a large amount of detection data is transmitted using the TDMA protocol. The detection data are firstly gathered within each array to array heads, and then, the heads send the data to the fusion center. In each array, the head allocates a time slot $T_{slot}$ for every member node.

**Figure 3:**
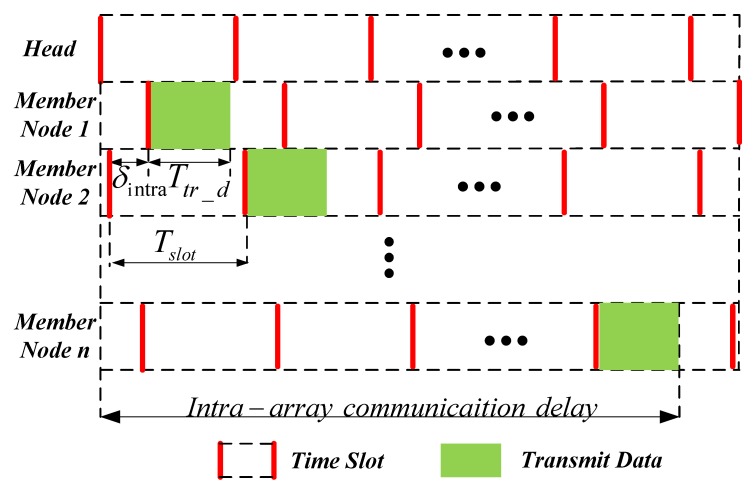
TDMA communication process.

Under the ideal situation without clock error, data collision is avoided by guaranteeing that every node can complete transmission in its own slot. However, as shown in Figure 3, different nodes have different understandings of the starting time of each slot due to clock errors $\delta_{intra}$ ($\delta_{intra}>0$). For any two nodes with neighboring time slots (for example, Member Nodes 1 and 2 in Figure 3), if no data collision happens, it satisfies:

(8) $$T_{tr\_d}+\delta_{intra}\leq T_{slot}.$$

where $T_{tr\_d}$ is the time to transmit a data packet. Let $l_{d}$ denote the length of every data packet from member nodes and *r* denote the sending rate; we have $T_{tr\_d}=\frac{l_{d}}{r}$.

Besides data collision, communication delay is another important metric of reliable communication. Since the head will forward the data to the fusion center only when it has received all the data from its member nodes, the intra-array communication delay $d_{intra}$ equals the communication delay of member node *n*. As shown in Figure 3, the communication delay of member node *n* is:

(9) $$d_{n^{th}}=\delta_{intra}+\left(n-1\right)T_{slot}+T_{tr\_d}\approx nT_{slot}.$$

Given the maximum tolerable intra-array communication delay $d_{\max1}$, we have:

(10) $$d_{intra}=nT_{slot}\leq d_{\max1}.$$

Combining (8) and (10), we get intra-array synchronization accuracy satisfying:

(11) $$0<\delta_{intra}\leq \frac{d_{\max1}}{n}-\frac{l_{d}}{r}.$$

After the heads receive all the member nodes’ data, they will forward the data to the fusion center. The length of each head’s data is $nl_{d}$. Suppose that there are *m* heads in our system, and the maximum tolerable communication delay is set to $d_{\max2}$ here, so the synchronization accuracy between heads (inter-array) must satisfy:

(12) $$0<\delta_{inter}\leq \frac{d_{\max2}}{m}-\frac{nl_{d}}{r}.$$

### 4.3. Demand of Data Fusion

After the detection data are sent to the sink node, we obtain the target’s position through intra-array fusion and inter-array fusion.

**Figure 4:**
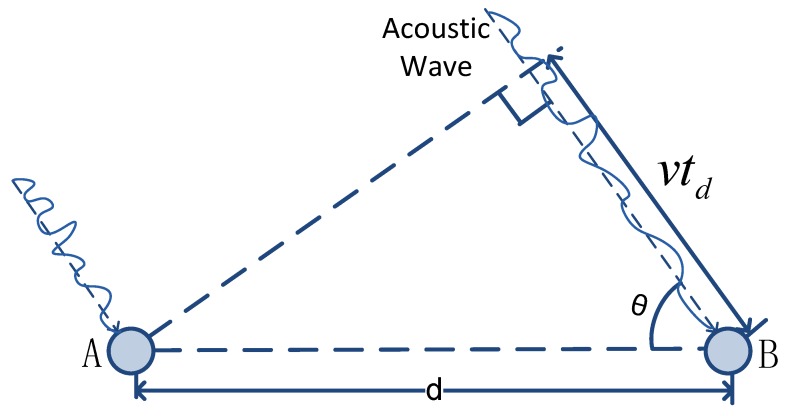
Acoustic wave arrives at two different nodes.

**Intra-array fusion:** We assume that acoustic wave arrives as a plane wave. As shown in Figure 4, node *A* and node *B* are two different nodes in the same array; the wave arrives at them at different times. The time difference of arrival is $t_{d}$. The acoustic wave propagates at a velocity of *v*. The distance between the nodes is *d*. Then, we can get the target’s direction with respect to the array by:

(13) $$\cos\left(\theta \right)=\frac{vt_{d}}{d}.$$

In practice, due to the clock error $\delta_{intra}$ ($\delta_{intra}>0$) between the two nodes, the recorded time difference of arrival becomes $t_{d}+\delta_{intra}$. Therefore, the obtained target’s direction is not accurate, and we have:

(14) $$\cos\left(\theta +\hat{\theta }\right)=\frac{v(t_{d}+\delta_{intra})}{d},$$

where $\hat{\theta }$ is the measurement error of the target’s direction.

For simplicity, we assume that $\hat{\theta }$ is small; thus, we have $cos(\hat{\theta })\approx 1$ and $sin\left(\hat{\theta }\right)\approx \hat{\theta }$. Then, by (13) and (14), we get:

(15) $$\hat{\theta }=-\frac{v\delta_{intra}}{d\sin\theta }<0,$$

where *d* is the distance between the nodes.

Given that the maximum tolerable measurement error of the target’s direction is ${\hat{\theta }}_{\max}>0$, we have $\hat{\theta }\geq -{\hat{\theta }}_{\max}$, i.e.,

(16) $$\frac{v\delta_{intra}}{d\sin\theta }\leq {\hat{\theta }}_{\max}.$$

Therefore, the intra-array synchronization accuracy satisfies:

(17) $$0<\delta_{intra}\leq \frac{{\hat{\theta }}_{\max}d\sin\theta }{v}.$$

**Figure 5:**
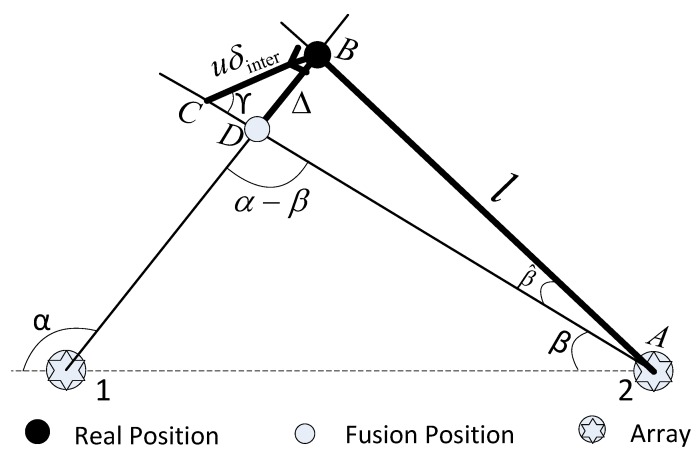
Method of obtaining the target’s position.

**Inter-array fusion:** Using direction information from several arrays, the sink node obtains the target’s position, as shown in Figure 5. The array is considered as a single point since the distance of any two arrays is much larger than the array’s size.

In the system, the detections from different arrays that have the same timestamp are fused to get the position of the target. Array 1, at local time $t_{A}=T$, detects the target and sends the detection data (containing timestamp *T*) to the sink node. By intra-array fusion, we obtain the target’s direction to Array 1, which is α. At the same time, array 2 also detects the target, but due to clock error $\delta_{inter}$ ($t_{1}-t_{2}=\delta_{inter}>0$) between the arrays, Array 2’s local time $t_{B}\neq T$, so it does not report detection data at this time. Instead, when its local time is $t_{B}=T$ and the target has moved $u\delta_{inter}$ (*u* is the target’s velocity), Array 2 sends its detection data (containing timestamp *T*) to the sink node. The target’s direction with respect to Array 2, which is β, is obtained. As we can see in Figure 5, there exists direction error $\hat{\beta }$; thus, the fusion position is different from the real position.

In triangle *ABC*, by the *law of sines*, we have:

(18) $$\sin\hat{\beta }=\frac{u\delta_{inter}\sin\gamma }{l},$$

i.e., the direction error $\hat{\beta }$ is due to the movement of the target.

In triangle *ABD*, by the *law of sines*, we get:

(19) $$\Delta =\frac{l\sin\hat{\beta }}{\sin(\alpha -\beta )},$$

i.e., the direction error $\hat{\beta }$ results in the localization error Δ.

Combining (18) and (19), we have:

(20) $$\Delta =\frac{u\delta_{inter}\sin\gamma }{\sin(\alpha -\beta )}.$$

Different from (19), Equation (20) tells us that the localization error is independent of the direction error $\hat{\beta }$ and the distance *l*. Because when *l* is larger, $\hat{\beta }$ will be smaller, and vice versa, $\hat{\beta }$ and *l* cancel each other out.

Given the maximum tolerable localization error $\Delta_{\max}$, we have $\Delta \leq \Delta_{\max}$. Therefore, the inter-array synchronization accuracy satisfies:

(21) $$0<\delta_{inter}\leq \frac{\Delta_{\max}\sin\left(\alpha -\beta \right)}{u\sin\gamma }.$$

In summary, the synchronization accuracy requirements of the target-tracking system are obtained in this section. We observe two notable features: *First*, the requirements are highly related to the system modes. *Second*, in the detection mode, the accuracy requirements of intra-array and inter-array synchronization are different. These features make the previous approaches, such as *RBS* [23] and *FTSP* [24], which focus on achieving high accuracy, not suitable for our target-tracking system. Therefore, it is necessary to design an energy-efficient synchronization protocol satisfying the different requirements of the target-tracking system.

## 5. Protocol Design

In this section, we present the design of *SASP*, an energy-efficient synchronization protocol for the target-tracking system, which mainly consists of three parts: synchronization in the sleep mode, synchronization in the detection mode, and synchronization in the transient state.

### 5.1. Synchronization in the Sleep Mode

In the sleep mode, the root node (fusion center) will broadcast maintenance packets periodically to other nodes to maintain the network status. Therefore, we can piggyback the synchronization information onto the maintenance packets. With the accuracy requirements in the sleep mode described in Section 4, the synchronization problem is formulated as follows:

(22) $$\begin{array}{cc} \mathrm{minimization} & E_{s}=f_{1}\left(\delta_{s}\right),\\ \mathrm{subject}\;\mathrm{to} & 0<\delta_{s}\leq \frac{rEP-e_{1}l_{m}L_{d}-re_{2}PL_{d}+e_{2}l_{m}L_{d}}{re_{1}L_{d}-re_{2}L_{d}}, \end{array}$$

where $E_{s}$ denotes the energy consumption of the synchronization process in the sleep mode.

We solve (22), the optimal accuracy is:

(23) $$\delta_{s}^{*}=\frac{rEP-e_{1}l_{m}L_{d}-re_{2}PL_{d}+e_{2}l_{m}L_{d}}{re_{1}L_{d}-re_{2}L_{d}}.$$

In general, the accuracy constraint can be satisfied using piggybacking. The synchronization period is N×P (*N* is a positive integer, and *P* denotes the network maintenance period). In fact, our evaluation results in Section 6 prove it.

### 5.2. Synchronization in the Detection Mode

We first check whether it is possible to piggyback inter-array or intra-array synchronization information using data packets. When a target appears, nodes surrounding it can detect it and send detection data to their heads, and then, these data are forwarded to the sink node. If we piggyback synchronization information onto these data packets, the head can get the time information from each member node; however, the member nodes cannot get a reference time to be synchronized. For faraway nodes that cannot detect the target, there is not even a data packet that needs to be sent. However, these nodes may also need to be accurately synchronized, since the target may move close to them in the future. Therefore, we can not synchronize the network by piggybacking in the detection mode.

With the accuracy requirements in the detection mode described in Section 4, the synchronization problem for inter-array synchronization is formulated as follows:

(24) $$\begin{array}{cc} \mathrm{Minimize} & E_{inter}=f_{2}\left(\delta_{inter}\right),\\ \mathrm{subject}\;\mathrm{to} & 0<\delta_{inter}\leq \frac{d_{\max2}}{m}-\frac{nl_{d}}{r},\\ & 0<\delta_{inter}\leq \frac{\Delta_{\max}\sin\left(\alpha -\beta \right)}{u\sin\gamma }, \end{array}$$

where $E_{inter}$ denotes the energy consumption of the inter-array synchronization process.

The synchronization problem for intra-array synchronization is formulated as follows:

(25) $$\begin{array}{cc} \mathrm{Minimize} & E_{intra}=f_{2}\left(\delta_{intra}\right),\\ \mathrm{subject}\;\mathrm{to} & 0<\delta_{intra}\leq \frac{d_{\max1}}{n}-\frac{l_{d}}{r},\\ & 0<\delta_{intra}\leq \frac{\theta_{\max}d\sin\theta }{v}, \end{array}$$

where $E_{intra}$ denotes the energy consumption of the intra-array synchronization process.

We solve (24) and (25), and the optimal accuracies are:

(26) $$\delta_{inter}^{*}=min\left(\frac{d_{\max2}}{m}-\frac{nl_{d}}{r},\frac{\Delta_{\max}\sin\left(\alpha -\beta \right)}{u\sin\gamma }\right),$$

and:

(27) $$\delta_{intra}^{*}=min\left(\frac{d_{\max1}}{n}-\frac{l_{d}}{r},\frac{\theta_{\max}d\sin\theta }{v}\right).$$

Note that $\delta_{inter}^{*}\neq \delta_{intra}^{*}$, and intra-array synchronization accuracy (clock error of nodes in the same array) is independent of inter-array synchronization accuracy (clock error of nodes of different arrays). Therefore, we design intra-array and inter-array synchronization, respectively.

**Intra-array synchronization**: For each array, the array head broadcasts a synchronization packet to its member nodes at the period of $T_{intra}$. Each member node receiving the packet then can synchronize with its array head.

**Inter-array synchronization**: Since each array is synchronized by its array head, we just need to synchronize the array heads to synchronize different arrays. Therefore, the sink node broadcasts a synchronization packet to all array heads in the period of $T_{inter}$, and then, each array head synchronizes with the sink node after receiving the synchronization packet.

It is obvious that our protocol is different from previous protocols, like FTSP [24], which synchronizes the whole network in the same period to achieve high network-wide accuracy. *SASP* saves energy consumption by providing different local and network-wide synchronization accuracy.

### 5.3. Synchronization in the Transient State

**Figure 6:**
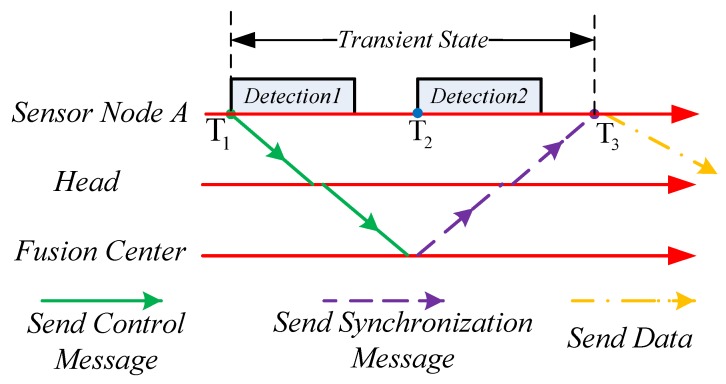
Node behaviors when the system transits from the sleep mode to the detection mode.

The synchronization accuracy in the sleep mode is lower than that in the detection mode. Thus, when targets appear, the system needs to be synchronized to higher accuracy; however, this takes time. As shown in Figure 6, when a target appears at $T_{1}$, node *A* detects it and immediately sends a *control message* to the sink. Then, node *A* starts detecting the target. After the sink node gets the *control message*, it broadcasts a synchronization message immediately to synchronize the network. At $T_{3}$, node *A* is synchronized. The time between $T_{1}$ and $T_{3}$ is called the *transient state*. In the *transient state*, the achieved synchronization accuracy cannot satisfy the requirements of the detection mode; thus, inaccurate localization happens. *SASP* takes this problem into consideration and solves it in two steps: (1) When node *A* receives the synchronization message at $T_{3}$, it calculates the difference between the reference time and its local time (the reference time minus the local time), which is $T_{offset}$. (2) Estimate the real starting time of each detection in the *transient state*. For example, in Figure 6, the real starting times of Detection 1 and Detection 2 are $T_{1}+T_{offset}$ and $T_{2}+T_{offset}$, respectively.

Overall, in the sleep mode, by piggybacking synchronization information onto network maintenance packets, we satisfy the accuracy constraint with low overhead. In the detection mode, we achieve precise network-wide and local synchronization to satisfy the inter-array and intra-array accuracy requirements. Furthermore, *SASP* eliminates the random delays of transmitting synchronization packets by using MAC-layer timestamping approach as in [22,24]. Moreover, compensation of clock drift [23,24] is employed to improve synchronization accuracy and prolong the synchronization period. Drift compensation in *SASP* is presented in Algorithm 1. We apply the linear model and use the latest offset value to estimate the drift.

**Algorithm 1** Drift compensation in the Sensor Array Synchronization Protocol (SASP).
1:**Initialization:** Drift compensation value comp[0]=0.2:**for**k=1; k++**do**3: Receive the synchronization packet of the $k^{\mathrm{th}}$ round of synchronization;4: Calculate the difference (offset) between the reference time and the node’s local time, $T_{offset}\left[k\right]$;5: $comp\left[k\right]=comp\left[k-1\right]+\frac{T_{offset}\left[k\right]}{T}$, where *T* is the synchronization period.6:
**end for**



## 6. Protocol Evaluation

### 6.1. Clock Model

Before evaluating our protocol, we first introduce the clock model. Nodes’ clocks are obtained from crystal oscillators, which could be significantly influenced by harsh environments, since nodes are deployed in the wild. In this paper, we adopt the WGN (White Gaussian Noise) random walk model [25,34] instead of the commonly-used constant-rate model to characterize the influence of the environment because the former model has higher uncertainty.

The clock model is described as follows:

(28) $$\theta \left[n\right]=\sum_{k=1}^{n}(1+d_{r}\left[k\right])\tau \left[k\right]+\theta \left[0\right]+w\left[n\right].$$

where *k* is the sample index, θ[n] is the node clock at the *n*th sample, τ[k] is the sampling period at the *k*th sample, and w[n] is the measurement noise, which follows a Gaussian random distribution with zero mean and variance $\sigma_{w}^{2}$. $d_{r}\left[k\right]$ is the clock drift at the *k*th sample, which is modeled as:

(29) $$d_{r}\left[n\right]=d_{r}\left[n-1\right]+\eta \left[n\right],$$

where η is the model noise with zero mean and variance $\sigma_{\eta }^{2}$.

### 6.2. Evaluation of SASP

We used simulation to evaluate the performance of *SASP*. The parameters used in the target-tracking system [9,28] are listed in Table 1. In our system, we used the CC2430 nodes. There were one fusion center and 40 sensor nodes, which were organized into eight arrays. In each array, there was one head node and four member nodes. The fusion center, head nodes, and member nodes form a hierarchical topology structure. Each node was powered by two batteries, which provided 1300 mAh of energy in total. Most of the time, there was no target, and the system worked in sleep mode. However, the system woke up every 120 s to maintain the network. The network maintenance packet was 40 bytes, sending at a rate of 250 Kbps. The piggybacked synchronization information was two bytes. The energy consumption of the CC2430 node in receiving, sending, and sleep states was 27 mA, 25 mA, and 0.9 µA, respectively [35]. For simplicity, in our simulation, the energy consumption was assumed to be 25 mA when the node was awake. Since most of the energy was consumed in the detection mode, we assumed that the energy supplied for the sleep mode was less than 10%, which was 100 mAh. A target may not be encountered for several months or even years. Thus, our system may sleep for a long time waiting for the target. The expected lifetime of the target tracking was set to be five years. However, if the target was encountered frequently, the system life would be much shorter. The target’s velocity was set to 20 m/s. Note that the traditional tracking systems [9,28] have a drawback that the tracking results are not good when the target and sensor arrays are in a line (θ or α−β is very small). In our system, this problem was solved because we could freely choose the appropriate nodes to form arrays to detect targets while satisfying $\theta >10^{\circ }$ and $\alpha -\beta >10^{\circ }$.

According to (7), the synchronization accuracy constraint in the sleep mode is:

(30) $$0<\delta_{s}\leq 5.4\;\mathrm{ms}.$$

In the detection mode, according to (11) and (17), the accuracy constraints of the intra-array synchronization are:

(31) $$\begin{array}{c} 0<\delta_{intra}<800\;\mu s,\\ 0<\delta_{intra}<178\;\mu s. \end{array}$$

According to (12) and (21), the accuracy constraints of the inter-array synchronization are:

(32) $$\begin{array}{c} 0<\delta_{inter}<9\;\mathrm{ms},\\ 0<\delta_{inter}<850\;\mu s. \end{array}$$

From (30)–(32), we can observe that the the accuracy constraints in the detection mode were much stricter than that in the sleep mode, and the constraint of the intra-array accuracy was much stricter than that of the inter-array accuracy. We also found that the accuracy was constrained by the demand of data fusion. However, during its trajectory, when the system network scale became larger, or the data length became longer, the constraints given by the demand of reliable transmission would be stricter, even stricter than the constraint given by data fusion.

According to (23), (26) and (27), the optimal synchronization accuracies are:

(33) $$\delta_{s}^{*}=5.4\;\mathrm{ms},$$

(34) $$\delta_{intra}^{*}=min\left(800\;\mu s,178\;\mu s\right)=178\;\mu s,$$

and:

(35) $$\delta_{inter}^{*}=min\left(9\;\mathrm{ms},850\;\mu s\right)=850\;\mu s.$$

The parameters of our clock model are also listed in Table 1. $\sigma_{w}$ = 15 µs and $\sigma_{\eta }$ = 10^−7^ were derived from [25,34]. Since we used the CC2430 node, the clock drift $d_{r}$ ranged from −40 ppm to 40 ppm [35]. The simulation results were statistical clock performances of over 1000-times the synchronization. Our performance evaluation mainly concentrated on two synchronization metrics:

- *Accuracy violation probability*: The probability of the achieved accuracy cannot satisfy the accuracy constraints (i.e., (30)–(32)). In this paper, the tolerable accuracy violation probability was set to 1%.
- *Average synchronization accuracy*: The synchronization accuracy averaged over all the runs for every pair of selected nodes (e.g., inter-array nodes, intra-array nodes).

#### 6.2.1. SASP in the *Sleep* Mode

**Figure 7:**
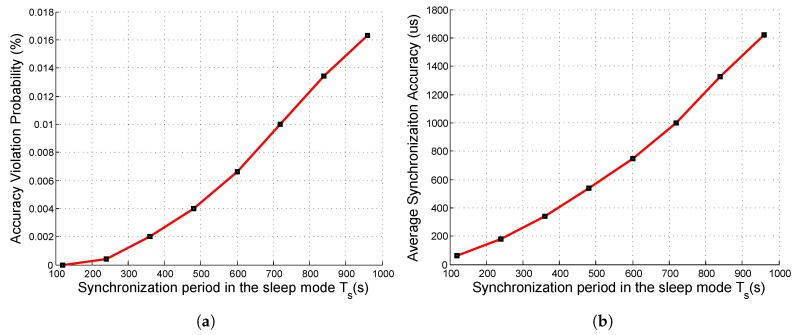
Synchronization metrics under different synchronization periods in the sleep mode: (**a**) accuracy violation probability; (**b**) average synchronization accuracy.

In the sleep mode, the synchronization bytes were piggybacked onto the network maintenance packets. Since the network maintenance period in the sleep mode was 120 s, and the synchronization period should be N×120 s (*N* is a positive integer). The simulation results are shown in Figure 7.

As shown in Figure 7a, when the synchronization period in the sleep mode $T_{s}$ was 720 s, the accuracy violation probability was about 1%, which is tolerable. Thus, by our simulation results, $T_{s}$ was set to 720 s in our system, and the corresponding intra-array average synchronization accuracy was 1 ms. Since the length of the piggybacked synchronization bytes was two bytes and the length of a network maintenance packet was 40 bytes, we obtained the overhead of piggybacking, which was $\frac{2}{40}\times \frac{120}{720}$ = 0.8%, which is negligible. Thus, *SASP* was effective in the sleep mode.

#### 6.2.2. SASP in the *Detection* Mode

**Figure 8:**
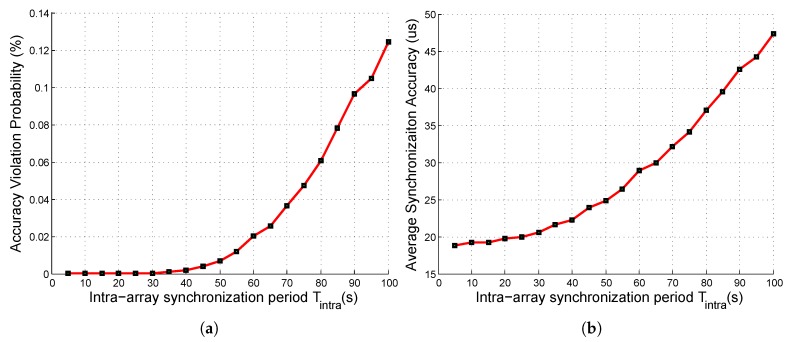
Intra-array synchronization metrics under different intra-array synchronization periods: (**a**) accuracy violation probability; (**b**) average synchronization accuracy.

*SASP* contains two synchronization in the detection mode: intra-array synchronization and inter-array synchronization. We first studied the intra-array synchronization. The simulation results are shown in Figure 8.

As shown in Figure 8a, when $T_{intra}$ was less than 30 s, the accuracy violation probability of intra-array synchronization was zero, i.e., the intra-array accuracy constraints in (31) were completely satisfied. When $T_{intra}$ was 50 s, the accuracy violation probability was about 1%, which is tolerable. However, if $T_{intra}$ became larger, the intra-array accuracy constraints could not be well satisfied. Thus, by our simulation results, the intra-array synchronization period $T_{intra}$ was set to 50 s in our system, and the corresponding intra-array average synchronization accuracy was 25 µs.

**Figure 9:**
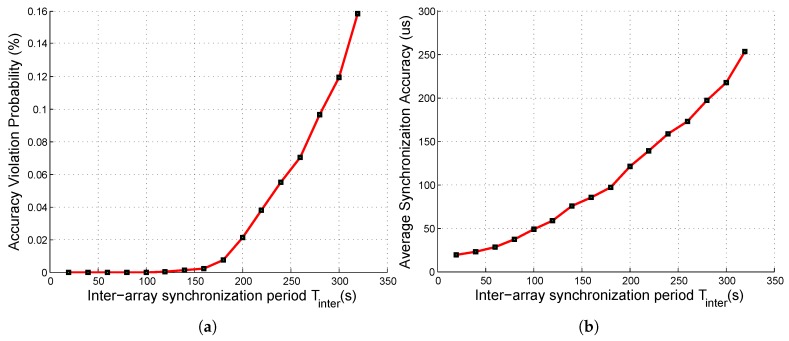
Inter-array synchronization metrics under different inter-array synchronization periods: (**a**) accuracy violation probability; (**b**) average synchronization accuracy.

After the arrays were synchronized by the array heads, we synchronized the array heads to synchronize different arrays. We set $T_{intra}$ to 50 s and varied $T_{inter}$, and we show the simulation results in Figure 9.

As shown in Figure 9a, when $T_{inter}$ was smaller than 160 s, the accuracy violation probability of inter-array synchronization was less than 1%, i.e., the inter-array accuracy constraints in (32) were satisfied. However, when $T_{inter}$ became larger, the violation probability was intolerable. Thus, the inter-array synchronization period $T_{inter}$ was set to 160 s in our system, and the corresponding inter-array average synchronization accuracy was 100 µs, which is lower than the intra-array one (25 µs).

From Figure 8 and Figure 9, we observe that the accuracy violation probability was more important than the average synchronization accuracy. For example, when $T_{intra}$ = 100 s, although the average synchronization accuracy (48 µs) was higher than the required (optimal) accuracy $\delta_{intra}^{*}$ (178 µs), the intra-array accuracy violation probability was 12%, which means the QoS was not guaranteed. Thus, *SASP* was designed to satisfy the accuracy constraints other than achieving high average accuracy, as in [22,23,24].

**Figure 10:**
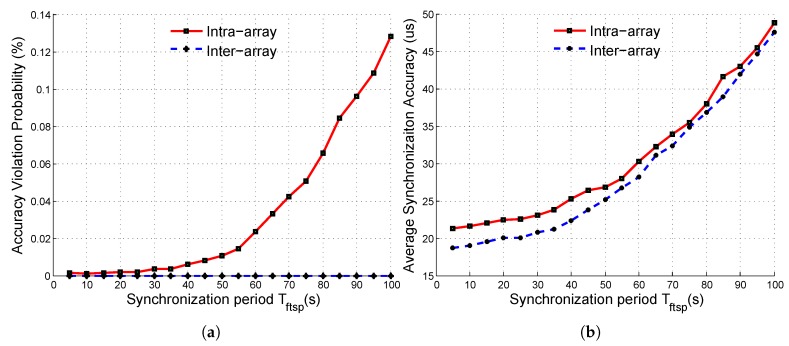
Synchronization metrics when using the Flooding Time Synchronization Protocol (FTSP) protocol. (**a**) accuracy violation probability; (**b**) average synchronization accuracy.

To further verify the effectiveness of *SASP*, we compared it with *FTSP, FTSP*. The simulation results of *FTSP* are shown in Figure 10. As shown in Figure 10b, the intra-array and inter-array synchronization accuracy were similar. That is because *FTSP* synchronized the network in the same period $T_{ftsp}$ and provided on average good synchronization between arbitrary nodes. However, in our system, the accuracy constraints of intra-array synchronization were stricter. Thus, as shown in Figure 10a, the intra-array synchronization accuracy would not satisfy its constraints as $T_{ftsp}$ became larger than 45 s, while the inter-array synchronization accuracy could still satisfy its constraints. Therefore, in order to satisfy both the inter-array and intra-array accuracy constraints, $T_{ftsp}$ should be shorter than 45 s in our system.

**Figure 11:**
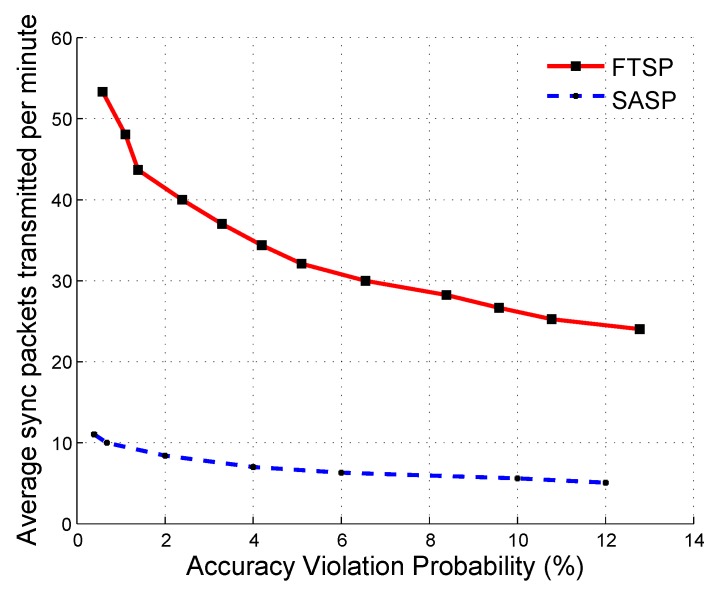
The average synchronization packets transmitted per minute (60 s) for SASP and FTSP under different accuracy violation probabilities.

To show the energy efficiency of *SASP*, we compared the energy consumption of *SASP* and *FTSP* under different accuracy violation probability. Since the energy consumption of transmitting one synchronization packet was almost the same in the two protocols, we used the number of synchronization packets transmitted per minute to indicate the energy consumption. The results are shown in Figure 11. As shown in Figure 11, *SASP* required less synchronization packets under all situations, so it was more energy efficient than *FTSP*. To guarantee the accuracy violation probability to be less than 1% (i.e., both (31) and (32) are satisfied), *SASP* required 10 synchronization packets per minute, while *FTSP* required 50. That is to say, using SASP saved 80% of the energy compared to using FTSP.

## 7. Conclusions

In this paper, we presented an energy-efficient time synchronization protocol for the target-tracking system in wireless sensor array networks, called *SASP*. To satisfy the synchronization accuracy requirements and save energy, *SASP* provided different synchronization accuracies according to the system mode and the network scope. Evaluation results demonstrated that: (1) *SASP* satisfied all the synchronization accuracy requirements; (2) the energy consumption of *SASP* was negligible in the sleep mode; (3) *SASP* was more energy efficient than *FTSP* in the detection mode. As part of our future work, we will apply our work to a few well-known protocols and systems. 

## Figures and Tables

**Table 1:** System constants and simulation parameters.

System Parameters	
Node’s energy consumption awake $e_{1}$ (mA)	25
Node’s energy consumption asleep $e_{2}$ (µA)	0.9
Energy supplied for the sleep mode *E* (mAh)	100
Operation period in the sleep mode *P* (s)	120
Length of a network maintenance packet $l_{m}$ (bytes)	40
Length of piggybacked synchronization bytes $l_{pg}$ (bytes)	2
Sending rate of wireless communication *r* (Kbps)	250
Expected lifetime of the target-tracking system $L_{d}$ (year)	5
Length of a detection data packet $l_{d}$ (bytes)	100
Number of nodes in every array *n*	5
Tolerable intra-array communication delay $d_{max1}$ (ms)	20
Number of heads in the system *m*	8
Tolerable inter-array communication delay $d_{max2}$ (ms)	200
Velocity of the acoustic wave in the air *v* (m/s)	340
Distance between nodes in the same array *d* (m)	20
Tolerable error of direction measurement ${\hat{\theta }}_{max}$ (degree)	1
Tolerable localization error $\Delta_{\max}$ (cm)	10
Target’s velocity *u* (m/s)	20
Clock model parameters	
$\sigma_{w}$ (µs)	15
$\sigma_{\eta }$	10^−7^
Clock drift $d_{r}$ (ppm)	−40–40
